# Bacterial pneumonia coinfection and antimicrobial therapy duration in SARS-CoV-2 (COVID-19) infection

**DOI:** 10.1093/jacamr/dlaa071

**Published:** 2020-08-25

**Authors:** Liam Townsend, Gerry Hughes, Colm Kerr, Mary Kelly, Roisin O’Connor, Eileen Sweeney, Catriona Doyle, Ruth O’Riordan, Ignacio Martin-Loeches, Colm Bergin, Ciaran Bannan

**Affiliations:** 1 Department of Infectious Diseases, St James’s Hospital, Dublin, Ireland; 2 Department of Clinical Medicine, School of Medicine, Trinity Translational Medicine Institute, Trinity College Dublin, Ireland; 3 Department of Pharmacy, St James’s Hospital, Dublin, Ireland; 4 Department of Intensive Care Medicine, Multidisciplinary Intensive Care Research Organization (MICRO), St James Hospital, Dublin 8, Ireland

## Abstract

**Background:**

Bacterial respiratory coinfection in the setting of SARS-CoV-2 infection remains poorly described. A description of coinfection and antimicrobial usage is needed to guide ongoing antimicrobial stewardship.

**Objectives:**

To assess the rate of empirical antimicrobial treatment in COVID-19 cases, assess the rate and methods of microbiological sampling, assess the rate of bacterial respiratory coinfections and evaluate the factors associated with antimicrobial therapy in this cohort.

**Methods:**

Inpatients with positive SARS-CoV-2 PCR were recruited. Antibiotic prescription, choice and duration were recorded. Taking of microbiological samples (sputum culture, blood culture, urinary antigens) and culture positivity rate was also recorded. Linear regression was performed to determine factors associated with prolonged antimicrobial administration.

**Results:**

A total of 117 patients were recruited; 84 (72%) were prescribed antimicrobial therapy for lower respiratory tract infections. Respiratory pathogens were identified in seven (6%) patients. The median duration of antimicrobial therapy was 7 days. C-reactive protein level, oxygen requirement and positive cultures were associated with prolonged duration of therapy.

**Conclusions:**

The rate of bacterial coinfection in SARS-CoV-2 is low. Despite this, prolonged courses of antimicrobial therapy were prescribed in our cohort. We recommend active antimicrobial stewardship in COVID-19 cases to ensure appropriate antimicrobial prescribing.

## Introduction

The SARS-CoV-2 (COVID-19) pandemic has placed an immense strain on healthcare systems. The importance and frequency of respiratory bacterial coinfection and need for concurrent antimicrobial therapy remains unclear. Concurrent bacterial or fungal infection rates in COVID-19 infection appear to be low.^1^^,^^2^ The rates are much lower than in patients admitted with influenza^3^ and also appear to be lower than for other coronaviruses.^4^ However, COVID-19 may be indistinguishable from bacterial respiratory tract infections at the time of presentation. As such, it is expected that empirical antimicrobial therapy is initiated pending pathogen identification.^5^^,^^6^ Rapid detection of coinfection is essential to the management of SARS-CoV-2 infection.^7^ There is a paucity of data regarding antimicrobial use, indication and duration in the setting of COVID-19. Prior to the COVID-19 pandemic, our institution provided twice-weekly ward visits by an antimicrobial stewardship (AMS) team, composed of infectious diseases clinicians, microbiologists and pharmacists. These were suspended during the pandemic due to reduced access to COVID-19 wards as well as secondment to COVID-related services for the AMS team. The objectives of this study were to assess the rate of empirical antimicrobial treatment in COVID-19 cases, assess the rate and methods of microbiological sampling, assess the rate of respiratory bacterial coinfection and evaluate the factors associated with antimicrobial therapy in this cohort in the absence of proactive AMS.

## Materials and methods

Ethical approval for this study was granted by the local research ethics committee (reference REC 2020-03). We recruited patients with a positive real-time PCR test for SARS-CoV-2 at our institution over a 2 month period (March–April 2020). We recorded the age and gender of all participants. Additionally participants were assessed for frailty, which was operationalized using Rockwood’s Clinical Frailty Scale (CFS) (range 0–7), chronic comorbidity count and polypharmacy assessment.^8–11^ The patients were followed over the course of their inpatient stay. Antimicrobial use, choice of antimicrobial and duration of therapy were recorded. Severity of COVID-19 infection was recorded. Individuals were classified as having severe disease if they required ICU admission or death occurred. Individuals who required ICU admission but were not admitted due to high likelihood of non-survival were considered as part of the severe group. This is a robust severity marker that has been used in previous COVID-19 studies.^12^ Peak C-reactive protein (CRP) and peak supplemental oxygen requirements were recorded for all participants.

The rate of diagnostic microbiological sampling was recorded. This included sputum culture (either self-expectorated or endotracheal aspirate), blood cultures and urinary antigen testing for *Streptococcus pneumoniae* and *Legionella pneumophila*. Bronchoalveolar lavage was not carried out on SARS-CoV-2-positive patients at our institution. PCR for alternative viral pathogens including influenza A and influenza B was not performed. The rate of culture positivity was recorded.

Median duration of antimicrobial therapy was calculated. Administration route was recorded, as well as IV-to-oral switch, with the median duration of both calculated. Between patient subgroups, normally and non-normally distributed quantitative data were compared using the Student’s *t*-test and Mann–Whitney *U*-test, respectively. Linear regression analysis was conducted to assess factors associated with antimicrobial duration. We tested the association of antimicrobial duration with culture positivity, markers of COVID-19 severity (need for critical care admission, peak CRP, peak supplemental oxygen requirement) and frailty markers (CFS, comorbidity count, co-medication count). The dates of positive microbiological cultures and positive SARS-CoV-2 PCR were compared and each case examined to assess whether infection could be deemed to be community acquired or nosocomial. Data were analysed using GraphPad Prism 8 and *P < *0.05 was considered statistically significant.

## Results

A total of 117 patients were admitted for management of COVID-19 infection: 74 were male and 43 were female. Thirty-four were admitted to ICU, while 11 were deemed in need of ICU-level care but were not managed at ward level due to a ceiling of care being in place. There were 17 deaths, giving an inpatient mortality rate of 14.5%. Seven patients remain inpatients due to ongoing rehabilitation needs. The median length of inpatient stay for those discharged was 12 days (range 2–60). Of the 117 patients, 95 (81%) were prescribed antimicrobials within 24 h of COVID-19 diagnosis. Eighty-four patients were treated for presumed lower respiratory tract infection (LRTI) (84/117, 72%), while 11 were treated for an alternative source of bacterial coinfection (11/117, 9%).

**Figure 1.**
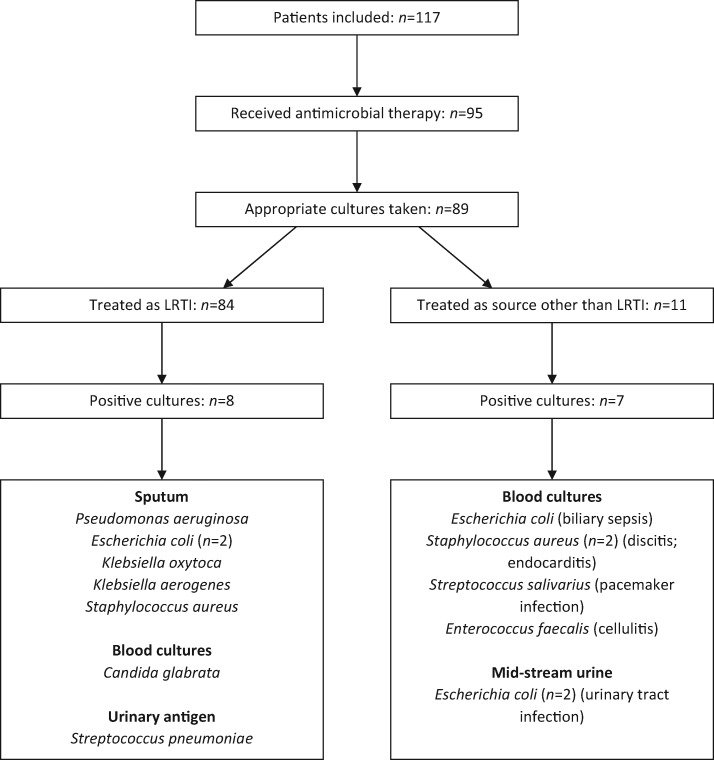
Patient pathway and microbiological isolates. All patients shown, with those receiving antimicrobials selected. Those treated as LRTI are shown on the left. The positive cultures are shown, including source of positive result. Those treated as source other than LRTI have their presumed source in parenthesis after the organism isolated.

Of the 95 patients treated for concurrent bacterial infection, 89 had at least one form of microbiological culture taken. No cultures were taken in the 22 patients who did not receive antibiotics. Positive cultures were found in 15 cases; however, 7 of these were found in patients receiving antimicrobial therapy with an indication other than respiratory tract infection. The organism identified and source of infection are shown in Table 1. Of the 84 patients treated empirically for respiratory tract infection (either community-acquired pneumonia [CAP], hospital-acquired pneumonia [HAP] or ventilator-associated pneumonia [VAP]), 79 (94%) had microbiological samples taken, with positive cultures in 8 patients. The samples taken were blood cultures (74/84; 88%), sputum samples (34/84; 40%) and urine samples for urinary antigen testing (27/84; 32%). On review of these samples, the identification of *Candida glabrata* in blood cultures was felt to represent infection of an indwelling venous catheter, while the remaining seven cultures were felt to represent respiratory tract infection. This gives a lower respiratory tract bacterial coinfection rate of 6% (7/117). The patient pathway and microbiological results are shown in Figure 1.

**Table 1.** Characteristics of treatment groups, antimicrobial choice and antimicrobial treatment duration

	Non-severe (*n *=* *44)	Severe (*n *=* *40)	Significance
Gender	male 29, female 15	male 29, female 11	ns
Age, median (range), years	66 (26–90)	66 (21–92)	ns
CFS, median (range)	3 (1–7)	3 (1–7)	ns
Antimicrobial choice (*n*)	cephalosporin (4)	cephalosporin (2)	
	amoxicillin/clavulanic acid (20)	amoxicillin/clavulanic acid (13)	
	piperacillin/tazobactam (18)	piperacillin/tazobactam (19)	
	meropenem (1)	meropenem (3)	
	anidulafungin (1)	aztreonam (1)	
	amoxicillin/clavulanic acid* *+* *clarithromycin (6)	levofloxacin (1)	
		linezolid (1)	
Antimicrobial duration, median (range), days	6 (2–14)	7 (1–14)	ns

Comparison of non-severe and severe respiratory infection groups. Demographics, antimicrobial prescription and antimicrobial treatment duration are shown. No significant difference between groups is noted.

ns, not significant.

Regarding the 84 patients treated empirically for respiratory bacterial coinfection, 78 received monotherapy. All treatment was initially given IV. An oral switch took place in only 34 cases. The median duration of IV therapy was 5 days (range 1–14), while the median duration of oral therapy was 3 days (range 1–4). The respiratory indications for treatment were CAP (*n *=* *50), HAP (*n *=* *32) and VAP (*n *=* *2). The median duration of treatment in the entire group was 7 days (range 1–14). Forty patients treated for respiratory tract infection met criteria for severe infection (death or requiring ICU admission). The demographics, antibiotic choice and duration of therapy in the non-severe and severe groups are shown in Table 1.

Factors associated with longer antimicrobial duration were investigated. Positive cultures were associated with longer duration of therapy (*P *=* *0.0041). Both peak CRP (*P *=* *0.0009) and peak supplemental oxygen requirements (*P *=* *0.026) were associated with increased duration of antimicrobial therapy. There were no associations of age, comorbidities or frailty markers (CFS, chronic comorbidity count or co-medication count) with duration of therapy.

## Discussion

Empirical antimicrobial therapy for bacterial LRTI was commenced in 72% of our cohort. Microbiological sampling was performed in the majority of these individuals, with blood cultures the most commonly taken sample. Sputum and urinary antigen testing were performed less frequently. The low rate of sputum testing is likely reflective of the non-productive nature of the classic COVID-19 cough, while urinary antigen testing has previously been reported to be carried out in less than 50% of hospitalized LRTI cases.^13^

The rate of concurrent bacterial LRTI in this cohort was 6%, which is reflective of those reported elsewhere.^1^^,^^2^ However, antimicrobial therapy was continued after the return of a positive SARS-CoV-2 PCR result and absence of microbiological evidence of bacterial infection in the majority of patients, with median duration of antimicrobial therapy of 7 days. It is advised that IV-to-oral switch is performed within 72 h in the absence of ongoing indication for parenteral therapy.^14^ The median duration of IV administration of antimicrobials in our study was 5 days, with only 34 patients undergoing oral switch. This was evident in the high levels of piperacillin/tazobactam prescribed in both severe and non-severe disease, despite our local guidelines reserving piperacillin/tazobactam for HAP. Furthermore, monotherapy was the predominant therapy, with only six patients receiving our local empirical antibiotic therapy of choice for CAP of amoxicillin/clavulanic acid and clarithromycin.

When factors associated with prolonged antimicrobial course were examined, elevated CRP and increased oxygen requirements were positively associated with duration. Age and frailty have previously been associated with increased antimicrobial usage in the general population.^15^ Interestingly, these factors were not associated with prolonged antimicrobial therapy in our study.

Our single-centre study has several limitations worthy of discussion. It is retrospective in nature, and therefore may be susceptible to selection bias. The treating physicians also varied across the patient group, which may result in differing practices in similar clinical situations. We would recommend that prospective studies with AMS intervention in a subgroup be carried out in the event of an ongoing or recurrent COVID-19 pandemic.

### Conclusions

This study reflects antimicrobial prescribing practices during the COVID-19 pandemic in the absence of an active AMS team. We demonstrate prolonged duration of antimicrobial treatment despite an alternative diagnosis and absence of evidence for bacterial coinfection. We suggest that AMS interventions are required in any ongoing COVID-19 infections, particularly focusing on discontinuing empirical therapy in the setting of negative cultures.

## Funding

This work was performed within the Irish Clinical Academic Training (ICAT) Programme, supported by the Wellcome Trust and the Health Research Board (Grant Number 203930/B/16/Z), the Health Service Executive, National Doctors Training and Planning and the Health and Social Care, Research and Development Division, Northern Ireland.

## Transparency declarations

None to declare.
